# Factors associated with high costs of patients with metabolic dysfunction-associated steatotic liver disease: an observational study using the French CONSTANCES cohort

**DOI:** 10.1186/s40842-023-00163-4

**Published:** 2024-04-25

**Authors:** Arnaud Nze Ossima, Angélique Brzustowski, Valérie Paradis, Bernard Van Beers, Catherine Postic, Cédric Laouénan, Stanislas Pol, Laurent Castéra, Jean-François Gautier, Sebastien Czernichow, Anais Vallet-Pichard, Etienne Larger, Lawrence Serfaty, Marie Zins, Dominique Valla, Isabelle Durand Zaleski

**Affiliations:** 1 DRCI- Health economics, Assistance Publique-Hôpitaux de Paris, Hôpital de l’Hôtel Dieu, 75004 Paris, France; 2 Université Paris Cité, INSERM, Centre de recherche sur l’inflammation, F-75018 Paris, France; 3 Université Paris Cité, Paris, France AP-HP, Hôpital Beaujon, 92110 Clichy, France Service Anatomie et cytologie pathologiques, INSERM, Centre de recherche sur l’inflammation, F-75018 Paris, France; 4 Radiology, AP-HP, Hôpital Beaujon, 92110 Clichy, France; 5 Université Paris Cité, INSERM, IAME UMR 1137, Paris, France, AP-HP.Nord, Hôpital Bichat, Département d’Epidémiologie Biostatistique et Recherche Clinique, Paris, France; 6 Université Paris Cité and Université Sorbonne Paris Nord, Inserm, IAME, F-75018 Paris, France AP-HP, Hôpital Bichat Service DEBRC, 75018 Paris, France; 7 Liver department, Hôpital Cochin-APHP, Université Paris Cité, Paris, France; 8 Hepatology department, Hôpital Beaujon, AP-HP, Université Paris Cité, INSERM UMR 1149, CRI, Clichy, France; 9 Université Paris Cité, Assistance Publique-Hôpitaux de Paris, Hôpital Lariboisière group and Inserm U1151, Service de diabétologie et d’endocrinologie – Centre Universitaire du Diabète et de ses Complications, Paris, France; 10 Université de Paris-Cité and Université Sorbonne Paris Nord, Paris, France, Assistance Publique-Hôpitaux de Paris (AP-HP), Service de Nutrition, Centre Spécialisé Obésité, Hôpital Européen Georges Pompidou, Paris, France, Centre of Research in Epidemiology and Statistics (CRESS-U1153), Inserm, INRAE, Paris, France; 11 Université Paris Cité, Liver department, Hôpital Cochin-APHP, Paris, France; 12 Université Paris Cité, Diabetology department, Hôpital Cochin-APHP, Paris, France; 13 Université de Strasbourg, Hepatogastroenterology Service, Hôpital Hautepierre, Hôpitaux Universitaires de Strasbourg 67000, Strasbourg, France, INSERM UMR_S938, Sorbonne Université, Paris, France; 14 UMS 11 Inserm, Versailles-Saint Quentin University, Versailles, France; 15 Service hépatologie, AP-HP, Hôpital Beaujon, 92110 Clichy, France; 16 Universite Paris Est Créteil, Assistance Publique-Hôpitaux de Paris, Service de Santé Publique, Henri Mondor-Albert- Chenevier, 94000 Créteil, France, Centre of Research in Epidemiology and Statistics (CRESS-U1153),Inserm, INRAE, Paris, France

**Keywords:** Healthcare costs, MASLD, NAFLD, Comorbidities, CONSTANCES, Claims data

## Abstract

**Background & aims:**

Despite its high prevalence in the western world metabolic dysfunction-associated steatotic liver disease (MASLD) does not benefit from targeted pharmacological therapy. We measured healthcare utilisation and identified factors associated with high-cost MASLD patients in France.

**Methods:**

The prevalent population with MASLD (including non-alcoholic steatohepatitis) in the CONSTANCES cohort, a nationally representative sample of 200,000 adults aged between 18 and 69, was linked to the French centralised national claims database (SNDS). Study participants were identified by the fatty liver index (FLI) over the period 2015–2019. MASLD individuals were classified according as “high-cost” (above 90th percentile) or “non-high cost” (below 90th percentile). Factors significantly associated with high costs were identified using a multivariate logistic regression model.

**Results:**

A total of 14,437 predominantly male (69%) participants with an average age of 53 ± SD 12 years were included. They mainly belonged to socially deprived population groups with co-morbidities such as diabetes, high blood pressure, mental health disorders and cardiovascular complications. The average expenditure was €1860 ± SD 4634 per year. High-cost MASLD cost €10,863 ± SD 10,859 per year. Conditions associated with high-cost were mental health disorders OR 1.79 (1.44–2.22), cardiovascular diseases OR 1.54 (1.21–1.95), metabolic comorbidities OR 1.50 (1.25–1.81), and respiratory disease OR 1.50 (1.11–2.00). The 10% high-cost participants accounted for 58% of the total national health care expenditures for MASLD.

**Conclusion:**

Our results emphasize the need for comprehensive management of the comorbid conditions which were the major cost drivers of MASLD.

**Graphical Abstract:**

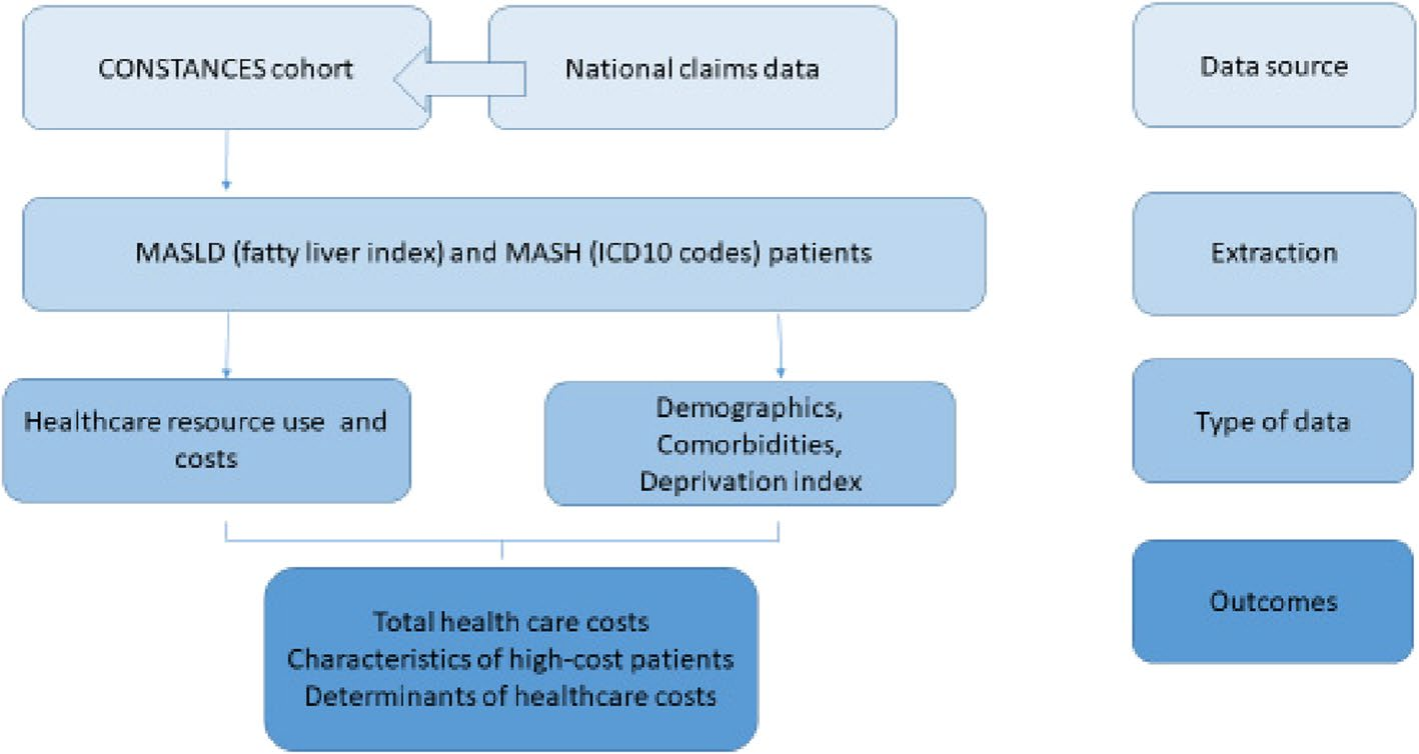

**Supplementary Information:**

The online version contains supplementary material available at 10.1186/s40842-023-00163-4.

## Highlights

• MASLD is a prevalent but low cost condition

• Over 50% of NAFLD participants belong to the more deprived population group

• The low average cost may result from limited awareness about complications

• Higher healthcare costs for NAFLD treatments are associated with comorbid conditions, in particular psychiatric disorders, diabetes, cardiovascular and respiratory diseases.

**Supplementary Information:**

The online version contains supplementary material available at 10.1186/s40842-023-00163-4.

## Lay summary

Metabolic dysfunction-associated steatotic liver disease (MASLD) is the most common chronic liver disease in European countries, affecting 4–50% of the European population. Confirmation of diagnosis requires liver biopsy which is an invasive procedure. We studied the healthcare costs of patients with MASLD in order to identify cost predictors and cost drivers. We found that patients cost on average €1860 per year. Conditions associated with high-cost were mental health disorders, cardiovascular diseases, metabolic comorbidities, and respiratory disease.

**Supplementary Information:**

The online version contains supplementary material available at 10.1186/s40842-023-00163-4.

## Introduction

Non-alcoholic fatty liver disease (MASLD) is the most common chronic liver disease in European countries, affecting 25.10% (20.55–30.28%) of the Western European population with a rapidly increasing prevalence [1–4]. In a study based on the CONSTANCES (CONSulTANts des Centres d’Examens de Santé) cohort, using the Fatty Liver Index (FLI), an algorithm based on body mass index, waist circumference, triglycerides and gamma glutamyl transferase, the estimated prevalence of MASLD in France was 18.2% [5].

MASLD is considered a “silent” disease, as many patients do not show specific symptoms, are not diagnosed and do not seek healthcare until they are at an advanced stage. In many cases, symptoms are attributed to metabolic comorbidities (including diabetes mellitus (DM), hyper-lipidaemia and dyslipidaemia, which are associated with increased risk of cardiovascular disease (CVD) and chronic kidney disease) commonly associated with MASLD. Confirmation of the diagnosis of MASLD is by liver biopsy, which is not routinely performed in the absence of approved therapies to treat diagnosed patients.

Given the increasing prevalence of MASLD, the economic burden is undoubtedly considerable despite the low cost per patient. In a Markov modelling study, the economic impact of MASLD in France was estimated at €784 per patient per year [6]. However, this study was built on several modelling assumptions including a 23% prevalence and expert opinion to estimate healthcare resource use. Circumventing these limitations, another study conducted in adults diagnosed with MASLD/ non-alcoholic steatohepatitis (MASH) from the French hospital claims database between 2009 and 2015 estimated annual hospitalization costs per patient at €7736 [7]. Although this study provided direct cost data, it included only patients with a hospital admission and therefore at more advanced stages of liver disease and comorbid conditions including cardiovascular or renal disease.

The clinical and economic impact of comorbidities among MASLD participants cannot be neglected because of the poor health outcomes and the multiple expenses incurred, either due to a higher prevalence of hospitalizations and clinic visits or due to an increase in the number of drugs used. The objectives of this study were to: 1) evaluate the use of healthcare resources by MASLD patients; 2) evaluate which factors are associated at a higher cost in MASLD patients.

## Methods

A prevalence-based cost study was performed using 2019 French health administrative databases.

### Database

The study sample was derived from the CONSTANCES cohort, a population-based prospective cohort study that included 220,000 volunteers aged from 18 to 69 years at 21 health examination centers throughout France between 2012 and 2020 [8]. Participants in the cohort were randomly selected within the National Health Insurance Fund beneficiaries. In France, all salaried and self employed workers—whether active or retired—and their families, are affiliated to the National Health Insurance Fund (“Caisse Nationale d’Asssurance Maladie des travailleurs salaries”, CNAMTS) which covers approximately 97% of the French population. At inclusion, a health examination was performed by a physician, and self-administered questionnaires with items on lifestyle, health status, medical history, socio-economic status, occupational exposures and lifetime employment history were completed by the eligible participants at home. Each participant visits 1) a health screening centre for a full assessment including a physical examination and laboratory tests; and 2) one of CONSTANCES’s recruitment centers for a comprehensive evaluation including a physical examination and laboratory tests. Health data from CONSTANCES cohort is linked at the individual level to healthcare reimbursement data recorded in the French national health insurance information system (SNDS). The SNDS contains the data on claims submitted for all healthcare resources: primary care (types and dates of procedures performed by private physicians, dentists, etc.; medical devices and associated services, reimbursed drugs, etc.); private and public hospitalizations; cash benefits (sick leave, disability pensions, workers’ compensation, occupational disease, or death benefits).

### Patient population

We studied the prevalent population of MASLD (including MASH) in participants in CONSTANCES, aged ≥18 years on December 31, 2019, identified by the fatty liver index (FLI), over the period 2015 to 2019, having claimed at least one healthcare reimbursement during the year 2019 and still alive as of December 31, 2019 [9].

### Patient characteristics

Factors assessed for association with healthcare utilization included demographics, socioeconomic characteristics (e.g. age, sex, educational level, social deprivation index), Charlson Comorbidity Index (CCI) and comorbidities [10]. Presence of comorbidities (56 treated diseases, episodes of care, chronic treatments) was identified by algorithms combining inpatient diagnoses international classification of diseases 10th revision (ICD-10) code, long-term disease code used by the social health insurance, pharmacy, laboratory tests and medical procedures reimbursement claims. The detailed methodology of the Diseases Mapping algorithms are publicly available in French [11]. Exclusion criteria or hierarchical rules apply to some algorithms, and some conditions are therefore mutually exclusive (e.g., acute ischemic heart disease is prioritized over chronic ischemic heart disease and, for a given location, currently treated cancer over history of cancer).

### Outcome

The total healthcare cost was calculated as the sum of medical claims over a one-year period. It included all claims submitted. The French national health insurance information system database records the total cost of each claim, regardless of the reimbursement rate applied. MASLD individuals were classified as high cost (above 90th of the total healthcare cost) or “non-high cost” (below 90th of the total healthcare cost [12]. Costs were expressed in 2022 euros.

### Statistical analysis

We described demographic, socioeconomic and clinical characteristics using means, and standard deviations for continuous variables, and frequency and percent for categorical variables. Patients were grouped in percentile of health care expenditures, high cost was defined as the 90th percentile and we used logistic regression to identify predictors of high cost. We first selected the variables corresponding to identified risk factors and complications (e.g. diabetes, hypertension, cardiovascular disease, mental health disorders) or homogeneous groups of conditions (e.g. inflammatory bowel diseases).

To combine highly correlated conditions within disease categories, we performed pairwise and multivariate correlation analyses. This procedure helps to prevent multicollinearity, without information loss. We also excluded conditions with very low explained variance (comorbidities with a prevalence < 0.1%). A logistic regression model with a logit link was used to identify the demographic, socioeconomic and clinical characteristics significantly associated with high costs, performed. On the demand side, we tested age, sex, comorbidities and socioeconomic characteristics; to describe the effect of supply or access to healthcare, we used the healthcare utilization of the previous year. Coefficients were exponentiated to express effects as rate ratio estimates. Variables for which a *p*-value lower or equal to 0.2 was observed in the bivariate analysis were included in the regression model. Models were generated iteratively using a forward building approach to ensure convergence could be attained. To generate the most parsimonious model, we excluded factors associated with a *p*-value greater than or equal to 0.05.

The adjusted odds ratios (ORs) were calculated and displayed with their respective 95% confidence intervals (95% CI).

All statistical analyses were conducted R statistical software version 4.1.3 (R studio).

## Results

### Study population and characteristics

**Fig. 1:**
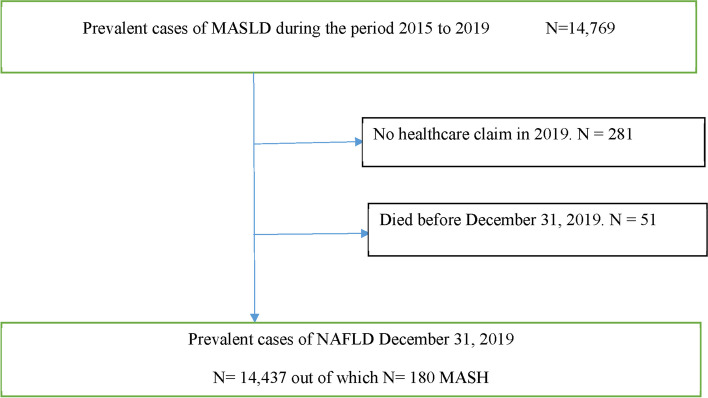
Patient selection flow chart. MASLD: Metabolic dysfunction-associated steatotic liver disease; MASH: Metabolic dysfunction-associated steatohepatitis

We identified 14,437 prevalent individuals with MASLD in 2019 (Fig. 1) out of a total population of 92,313 (after excluding subjects with a history of chronic viral hepatitis or excessive alcohol consumption. This assessment made at the baseline recruitment visit and allow to avoid misclassification of MASLD diagnosis.) or 15.6%. The average age (mean ± standard deviation) was 53 ± 12 years; 39% were aged > 58 years. Of the total individuals, ~ 69% were males, and CCI scores were 0.8 ± 1.3. Characteristics of MASLD individuals are shown in Table 1.

**Table 1:** Characteristics and chronic comorbidities of MASLD and MASH individuals

*N* = 14,437	
Male sex n (%)	9911 (68.6)
Mean age in 2018 (SD)	53 (12)
**Social Deprivation index, n (%)**	
1st quartile (least deprived)	1879 (13)
2nd quartile	2481 (17.2)
3rd quartile	2838 (19.7)
4th quartile	3354 (23.2)
5th quartile (most deprived)	3885 (26.9)
Educational level, n (%)	
No diploma	621 (4.3)
College, Certificate of primary or secondary education, GCE or A level	4690 (32.5)
high school	2495 (17.3)
higher education	6416 (44.4)
Other	215 (1.5)
Charlson Comorbidity Index	0.8 (1.3)
BMI at inclusion, Mean (SD)	31.5 (4.4)
> 30 n (%)	8377 (58.0)
Waist circumference at inclusion, Mean (SD)	103.9 (9.5)
**Comorbidities in 2018, n (%)**	
Cardio metabolic disease: hypertension, hyperlipidemia, diabetes	4765 (33.0)
mental health disorders	1839 (12.7)
Cardiovascular diseases and stroke	1036 (7.2)
Respiratory disease	854 (5.9)
Cancer	307 (2.1)
HIV infection or AIDS	31 (0.2)
Chronic inflammatory disease	261 (1.8)
Neurodegenerative disease	141 (1.0)

*SD* standard deviation

**Fig. 2:**
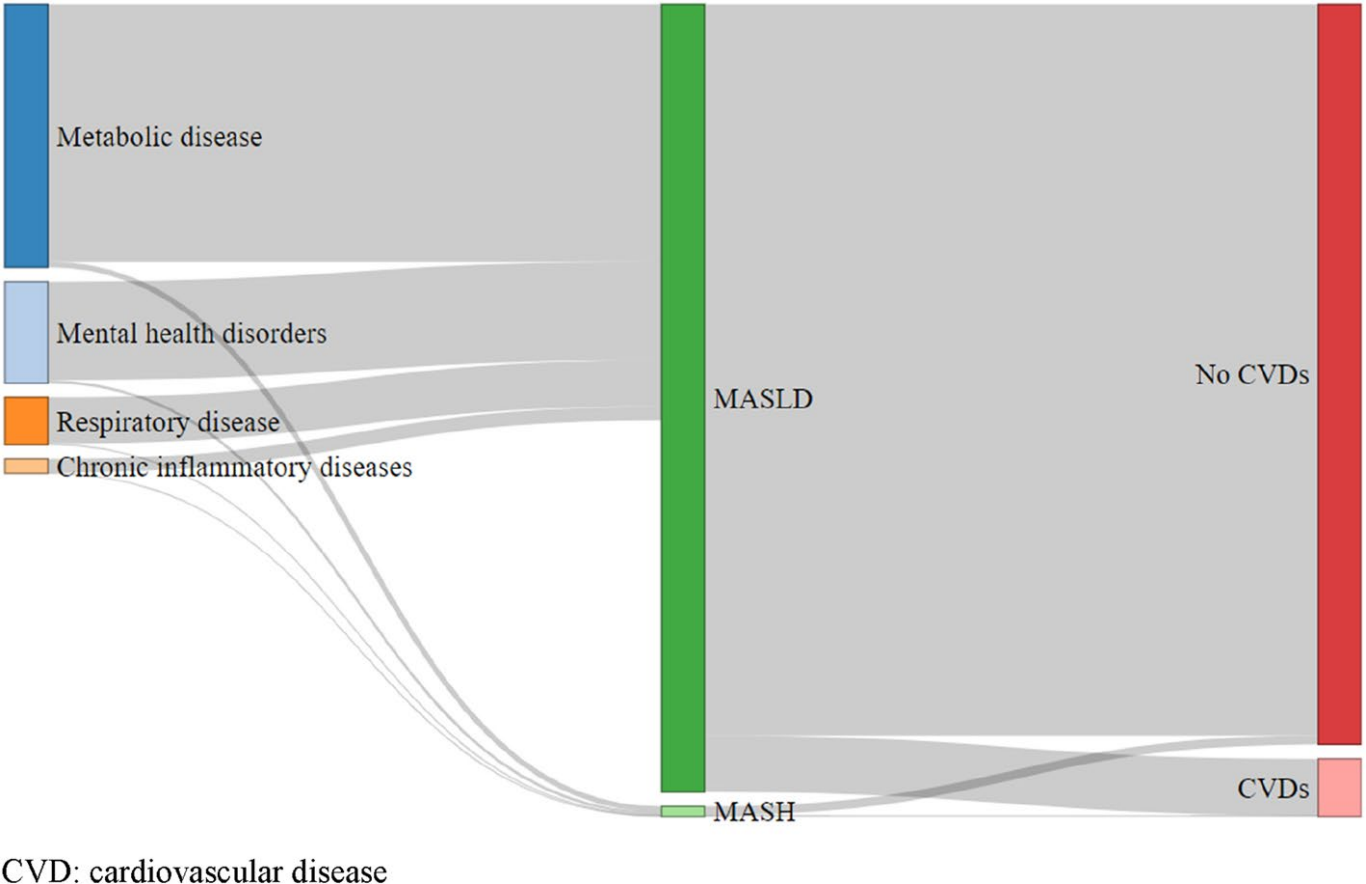
Prevalence of risk factors and complications in the MASLD/ MASH population of the CONSTANCE cohort. *N* = 14,437 participants, including 180 MASH patients. CVD: cardiovascular disease

Half of the MASLD individuals belonged to the two worst deprivation quintiles, despite the fact that over 60% of the population reported high school or university education. Metabolic risk factors and diabetes were the most prevalent comorbidities, followed by mental health disorders. Figure 2 describes the association between MASLD/ MASH and risk factors/ cardiovascular complications. While not a longitudinal study, it shows the frequent association of MASLD with metabolic disease and mental health disorders. We also examined the combinations of comorbidities and risk factors (Fig. S2) and found that the most frequent were metabolic and metal health disorders, followed by cardiovascular and respiratory disease.

### Healthcare costs in participants with MASLD

The mean healthcare expenditure per participant with MASLD was €1860 in 2019 (Table 2). Hospitalization represented the leading expenditure item with a mean of €673 representing 36% of all healthcare expenditure. Only 27% of MASLD individuals were hospitalised with primary diagnoses of diabetes, cardiovascular disease or mental health disorders. Of note, the mean length of stay was 0.6 ± 6 days. Pharmacy was also a substantial expenditure driver, representing 21% of all healthcare expenditure for 94% of MASLD patients. More specifically, about 35% of individuals with MASLD were treated for hypertension, 21% for dyslipidaemia, 15% for respiratory disease, and 9% for diabetes. Medical fees and paramedical visits accounted for 25% of healthcare expenditure (Table 2). 92% of patients visited the general practitioner in 2019, with a mean of 5 ± 4 visits. Nurses and physiotherapists were the main paramedical professionals consulted, by 46 and 32% of patients, respectively. Medical devices’ expenditure was driven by 10% of patients receiving ventilation at home including continuous positive airway pressure and oxygen therapy. Only 4 patients out of the 14,437 population had a procedural code for liver biopsy during the 2015–2019 period. For a total adult population of 53 million in France the estimated total healthcare cost was 15,7 billion €.

**Table 2:** Healthcare expenditure of individuals with MASLD in 2019 in €

	MASLD individuals *N* = 14,437 (%)	Costs € (SD)
General practitioner consultations	13,316 (92%)	104 (106)
Consultations with other professionals *	12,171 (84%)	285 (583)
Practice nurse consultations	6670 (46%)	20 (175)
Other paramedical care	4692 (32%)	86 (261)
Dental care	8373 (58%)	74 (137)
Laboratory tests	10,308 (71%)	71 (153)
Medical devices	6930 (48%)	144 (591)
Audio prostheses	318 (2%)	4 (37)
Drugs	13,571 (94%)	397 (1713)
Transportation		37 (367)
Hospital care	3898 (27%)	673 (3244)
Total healthcare cost		1860 (4634)

*SD* standard deviation

*Details of other professionals are in Table S1

### Characteristics of high-cost and non-high cost patients

Among MASLD individuals, 1444 were identified as being above the 90th percentile of the cost distribution (more than €4086 per year). Differences found between high cost and non-high cost individuals are described in Table 3. High cost individuals were older, more frequently female, and with multiple comorbidities, mostly metabolic (49.2% vs. 31.2%), mental health disorders (27.1% vs. 11.1%), cardiovascular diseases and stroke (19.3% vs. 5.8%), and respiratory disease (12.9% vs. 5.1%). No differences were found between high and non-high costs regarding social deprivation and education level. High-cost patients spent an average 5.6 days in the hospital, while non-high cost patients were barely hospitalized.

**Table 3:** Characteristics of high-cost and non-high cost MASLD individuals

	High cost 1444 (10%)	Non-high cost 12,993 (90%)
Sex, n (%)		
Female	543 (37.6)	3983 (30.7)
Mean age in 2018 (SD)	57.1 (11.2)	52.5 (12)
> 58 years (%)	791 (54.8)	4842 (37.3)
Social Deprivation index, n (%)		
1st quartile (least deprived)	189 (13.1)	1690 (13)
2nd quartile	234 (16.2)	2247 (17.3)
3rd quartile	285 (19.7)	2553 (19.)
4th quartile	319 (22.1)	3035 (23.4)
5th quartile (most deprived)	417 (28.9)	3468 (26.7)
Educational level, n (%)		
No diploma	82 (5.7)	539 (4.1)
College, Certificate of primary or secondary education, GCE or A level	598 (41.4)	4092 (31.5)
high school	243 (16.8)	2252 (17.3)
higher education	500 (34.6)	5916 (45.5)
Other	21 (1.5)	194 (1.5)
Charlson Comorbidity Index	1.3 (1.9)	0.7 (1.0)
Comorbidities in 2018, n (%)		
BMI at inclusion, Mean (SD)	32.6 (5.1)	31.4 (4.4)
> 30 n (%)	951 (65.9)	7426 (57.2)
Waist circumference at inclusion, Mean (SD)	106.6 (10.6)	103.6 (9.4)
Cardio metabolic comorbidities	710 (49.2)	4055 (31.2)
Cardiovascular diseases and stroke	279 (19.3)	757 (5.8)
Mental health disorders	391 (27.1)	1448 (11.1)
Respiratory disease	186 (12.9)	668 (5.1)
Chronic inflammatory disease	120 (8.3)	141 (1.1)
Average length of hospital stay (days), (SD)	5.6 (18)	0.1 (0.5)

*SD* Standard deviation

### Distribution of healthcare expenditure

Table 4 shows the cost distribution for patients in the high cost and non-high cost groups. The total mean annual cost was €859 in the non-high cost and €10,863 in the high-cost group or a 12.6-fold increase. Hospital cost contributed to ∼51% of total cost in high-cost vs 16% in non-high cost. The 10% most expensive individuals incurred 58% of total MASH expenditures.

**Table 4:** Healthcare expenditure of high-cost and non-high cost MASLD individuals in 2019 (€)

Mean (standard deviation)	Total *N* = 14,437	High cost 1444 (10%)	Non-high cost 12,993 (90%)
General practitioner consultations	104 (106)	214 (166)	92 (89)
Consultations with other professionals	285 (583)	1209 (1391)	183 (239)
Practice nurse consultations	20 (175)	141 (529)	7 (32)
Other paramedical care	86 (261)	330 (603)	59 (166)
Dental care	74 (137)	100 (188)	74 (137)
Laboratory tests	71 (153)	269 (380)	49 (72)
Medical devices	144 (591)	824 (1634)	68 (186)
Audio prostheses	4 (37)	5 (44)	3 (37)
Drugs	397 (1713)	2261 (4963)	190 (308)
Transportation	37 (367)	305 (1046)	7 (138)
Hospital care	673 (3244)	5509 (8825)	136 (391)
Total healthcare cost	1860 (4634)	10,863 (10,859)	859 (866)

### Factors associated with high-cost

The results of the bivariate logistic regression analysis are presented in Table S3. Based on these results, we included the following variables, presenting a *p*-value was lower than 0.2, in the multivariate logistic regression: sex, age, HC in 2018, Charlson Comorbidity Index score, metabolic comorbidities, cardiovascular diseases and stroke, mental health disorders, cancer, respiratory disease, HIV infection or AIDS, chronic inflammatory disease, and neurological and neurodegenerative disease.

Table 5 presents the multivariate logistic regression analysis results. After adjustment for other independent variables, of the comorbidities with a prevalence ≥5%, the highest odds for being a high-cost patient were associated with cardiovascular diseases and stroke (OR = 1.59, 95% CI: 1.25–2.03, *p* < 0.001), diabetes and other cardiovascular risk factors constitutive of metabolic syndrome (OR = 1.47, 95% CI: 1.22–1.78), respiratory disease (OR = 1.48, 95% CI: 1.10–1.98), and mental health disorders (OR = 1.77, 95% CI: 1.43–2.20); prostate cancer and colonic cancer were not selected because of their low prevalence. Additionally, the historical /supply effect measured by the health care utilization during the previous year had the highest impact, participants who were high users of healthcare in 2018 remained high users in 2019 (OR = 5.80 95% CI: 4.80–7.03). On the contrary, demographic characteristics did not have a significant impact on high-cost. While belonging to the most deprived population groups was associated with the risk of MASLD (Table 1), deprivation was no predictive of high cost, which might point to reduced access to care in this population. Patients in the two most deprived deciles had the same resource use as the average, both in terms of ambulatory and hospital care (Table S2).

**Table 5:** Multiple logistic regression analysis for high cost MASLD individuals

Risk factor	Adjusted odds ratio (95% CI)
High cost in 2018 (ref: non high cost in 2018)	5.80 (4.80–7.03)
BMI	1.04 (1.02–1.06)
Charlson Comorbidity Index score	1.29 (1.20–1.38)
Metabolic comorbidities	1.47 (1.22–1.78)
Cardiovascular diseases and stroke	1.59 (1.25–2.03)
Respiratory disease	1.48 (1.10–1.98)
Chronic inflammatory disease	4.12 (2.75–6.19)
Mental health disorders	1.77 (1.43–2.20)

## Discussion

### Main results and interpretation of the findings

We investigated the healthcare costs of individuals with MASLD and examined the characteristics of high-cost patients. MASLD is a high-prevalence but low-cost condition due in part to the lack of specific diagnostic tests and treatments, which leaves the patient population largely unattended. In our analysis of a representative sample of the French population, we found a prevalence of 15.6% and an average yearly cost of 1860€ (2676 US$), which is lower than the average yearly per capita healthcare expenditures in the French, or EU 27 populations (from €5000 in Germany to just below €2000 in Greece) and lower than the average cost of diabetic patients in France (2300€). Patients were treated by their general practitioners (92% had a consultation) and specialists, high-cost patients were referred to specialists and admitted to hospitals, but that may be for any of their other conditions. In this French population, the costs of laboratory tests was low (71€ per patient per year), which suggests no excessive use of liver function tests or other procedures: this might be the result of either good compliance with guidelines, or ignorance altogether of the disease [13].

The linkage with the national claims database provided quasi certainty that the diagnosis of MASLD was nearly never confirmed by a liver biopsy. We confirmed that high cost MASLD was associated with metabolic syndrome and a number of comorbid conditions, including mental health disorders [13]. In terms of other comorbidities, cardiovascular diseases, metabolic comorbidities, and respiratory diseases represented the most prevalent conditions. The 10 % most expensive patients with MASLD and other comorbid conditions incurred 58% of healthcare expenditures which is in accordance with the published range of 55–77% for high cost patients in general [14].

The drivers of high costs were hospital admissions and drugs; part of the utilization may be explained by a supply-induced demand or failure to coordinate the care for shared risk factors between diabetes, cardiovascular disease, respiratory disease and mental health disorders. It is however impossible to separate legitimate from overuse of healthcare during a short follow up. Symmetrically, while we identified that patients in the most deprived groups had the highest risk of MASLD, we did not find association between deprivation and use of healthcare services. The simplest explanation on the supply side is the limited access to specialized care in this population, or lack of diagnostic awareness among general practitioners, absence of coordinated care pathways, or ‘one stop shops’ which could manage the risk factors and comorbidities. On the demand side, we saw that nearly all patients attend GP consultations and other health professionals, and use medications, which is in a way reassuring as it means some degree of health literacy and trust in the healthcare system.

### Comparison with other studies

Our results align well with prior studies in the USA and Europe where the low cost of diagnosis and treatment of MASLD have been reported on population samples [15, 16]. The prevalence of MASLD reported in a previous study based on the CONSTANCES cohort after adjustment to ensure full representativeness of the French adult population was 18.2%, lower than the 25% worldwide estimate [5]. The total healthcare costs estimated at 15.7 billion € by our study also come close to the estimated 11.4 € billion in the model-based study of MASLD costs by Younoussi with 2015 cost data [6]. Regarding estimates for the MASH population, our results align well with prior studies in the USA and Europe where the low costs of diagnosis and treatment have been reported on population samples [15, 16]. We found a pattern of resource utilization of general practitioners and specialists consistent with the results of the National Health and Wellness Survey, albeit lower as can be expected for MASLD vs MASH patients [17]. Lower costs of € 699–771 for healthcare were reported for the MASH population in France, based upon prevalence estimates and literature data [16]. This was unexpected because the CONSTANCES cohort identifies also patients who are not diagnosed and therefore not actively monitored, however the cost of illness study by Schattenberg et al. estimated a ‘pure’ cost for MASH, without the additional resource use unrelated to the liver disease such as the metabolic syndrome and MASH complications, which affect roughly half of the MASLD population in our study (Fig. 2) [18].

### Strengths

These results on the high prevalence of MASLD, combined with our data on the drivers of high resource utilization suggest the need to better identify and monitor patients requiring the more expensive care, a group likely to include patients with significant comorbidities [13]. We also confirmed that MASLD affects predominantly low income patients [19]. However, being in the two most socially deprived quintiles did not result in fewer consultations with healthcare professionals or lower use of hospital care, possibly because the use of healthcare resource is already low. The fact that cardiovascular diseases, metabolic comorbidities and respiratory disease were strong predictors of high-cost is consistent with the pathology of MASLD. These comorbidities are common resource-intensive conditions and hare risk factors. Similarly, psychiatric disorders were significantly more common in the high-cost group. Another important finding is the recurrence of high costs in the same cluster of patients, which could allow targeting a group of persistent high-healthcare users for specific interventions on the comorbidities and the care pathways.

### Limitations

The major limitation of this study was the potentially existing selection bias. Participants selected in the CONSTANCES cohort are volunteers whose characteristics are different from the general population, particularly in terms of education and lifestyle (despite adjustment on demographic characteristics). Better lifestyle in CONSTANCES participants lead to a lower prevalence of metabolic diseases and, therefore, to a loss of power in statistical measures of association. Identification of MASLD patients used the fatty liver index, in the absence of a better marker and in particular the near total absence of liver biopsies. However, the diagnostic performance of this surrogate marker has been re assessed by a meta analysis, with a sensitivity 0.67 (CI 95% 0.62, 0.72) and specificity 0.78 (CI 95% 0.74, 0.83), it was considered the marker with highest diagnostic accuracy for MASLD [20]. Patients were not extracted from the CONSTANCES database using the newer cardiometabolic risk factors that are now part of the MAFLD definition [21]. Another limitation was that the relationships between clinical characteristics and medical costs does not allow causal interpretation due to the observational nature of data. This cost study was undertaken on a French population, which limits its external validity outside Europe (in the USA for example), but not inside Europe, as shown by the GAIN study [16]. Finally, our cost calculations excluded non-medical direct and indirect costs, which have been shown to amount to roughly the same as medical costs [16].

## Conclusion

The objective of the QUID MASH (Quantitative imaging in diabetic non-alcoholic steatohepatitis) project was to advance better care pathways for patients with MASLD and MASH, by understanding their unmet needs. We found that a majority of patients with MASLD do not have biopsies and therefore no diagnostic confirmation.

Metabolic and mental health comorbid conditions drove the use of healthcare resources. Given the lack of targeted pharmacological therapy for MASLD, the overall management of the MASLD patients requires interventions that holistically address the need of patients with multimorbidity risk factors, such as lifestyle services, educational programmes, and obesity programmes, or increased MASLD pathway capacity [19]. Regular physical activity has been shown to reduce the risk of developing MASLD and should become part of the integrated patient management particularly in view of the possible prescription and reimbursement of supervised formal exercise programs [20].

### Supplementary Information

**Additional file 1.**

## Data Availability

The data that support the findings of this study are available from the corresponding author upon reasonable request.

## Abbreviations

CONSTANCES: CONSulTANts des Centres d’Examens de Santé; patients recruited in primary care centers
FLI: Fatty Liver Index
QUID-NASH: Quantitative imaging in diabetic non-alcoholic steatohepatitis
SNDS: système national des données de sante; French national health claims data base
