# Supplementary material for: Factors associated with high costs of patients with metabolic dysfunction-associated steatotic liver disease: an observational study using the French CONSTANCES cohort
Source: Clin Diabetes Endocrinol. 2024 Apr 25;10:9. doi: 10.1186/s40842-023-00163-4 (PMC11044468; doi:10.1186/s40842-023-00163-4)

Table S1

Consultations with health professionals for MASLD patients in 2019

|  | MASLD patients  N = 14,437 (%) | Costs € (SD) |
| --- | --- | --- |
| Consultations with other professionals | 12,171 (84%) | 285 (583) |
| Radiology | 7,664 (53%) | 52 (86) |
| Surgery | 3,254 (22%) | 50 (179) |
| Cardiology | 3,102 (21%) | 38 (160) |
| Anaethesiology | 2,347 (16%) | 24 (91) |
| Ophtalmology | 5,746 (40%) | 21 (71) |
| Psychiatry | 612 (4%) | 15 (37) |
| Gastro-enterology and hepatology | 1,459 (10%) | 13 (53) |

Table S2

Healthcare resource utilization among the two most deprived quintiles (Q4 and Q5) of the MASLD population: total care expenditures in 2019 (€)

| Mean (Standard deviation) | Q4  N = 3,354 | Q5  N = 3,885 |
| --- | --- | --- |
| General practitioner consultations | 104 (108) | 111 (107) |
| Consultations with other professionals | 278 (594) | 286 (635) |
| Hospital care | 634 (2,783) | 707 (3,054) |
| Total healthcare cost | 1,800 (4,455) | 1,937 (5,067) |

Table S3

Univariate logistic regression analysis

| Risk factor | Odds ratio (95% CI) |
| --- | --- |
| Sex (ref: Male) | 1.36 (1.22-1.53) |
| Age (ref: <= 58 years) | 2.04 (1.83-2.28) |
| HC in 2018 (ref: NHC in 2018) | 14.92 (13.14-16.94) |
| Charlson Comorbidity Index score | 1.40 (1.31-1.48) |
| Comorbidities |  |
| Cardio Metabolic comorbidities | 2.13 (1.91-2.38) |
| Cardiovascular diseases and stroke | 3.87 (3.33-4.50) |
| mental health disorders | 2.96 (2.60-3.36) |
| Cancer | 3.91 (3.03-5.02) |
| COPD | 2.73 (2.29-3.24) |
| HIV infection or AIDS | 133.12 (40.09-824.27) |
| Chronic inflammatoiry disease | 8.26 (6.43-10.60) |
| Neurogenerative diseases | 4.04 (2.78-5.76) |

Figure S1

Associations between risk factors and comorbidities in the MASLD population (numbers represent the number of participants with the risk factors / comorbidities) in the population of 14,437 participants.


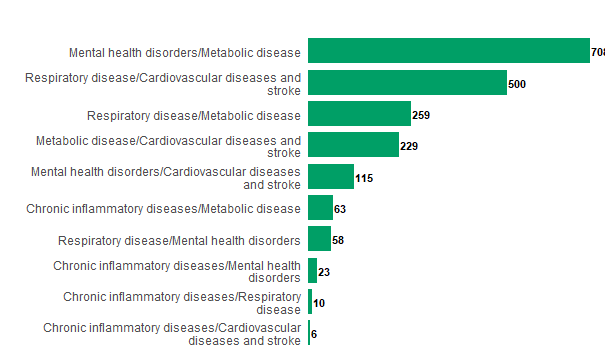

Supplement: Supplementary file 1 — Additional file 1. [file 40842_2023_163_MOESM1_ESM.docx]
